# Intestinal translocation of enterococci requires a threshold level of enterococcal overgrowth in the lumen

**DOI:** 10.1038/s41598-019-45441-3

**Published:** 2019-06-20

**Authors:** Cristel Archambaud, Aurélie Derré-Bobillot, Nicolas Lapaque, Lionel Rigottier-Gois, Pascale Serror

**Affiliations:** Micalis Institute, INRA, AgroParisTech, Université Paris-Saclay, 78350 Jouy en Josas, France

**Keywords:** Pathogens, Bacterial pathogenesis

## Abstract

Enterococci are subdominant members of the human gastrointestinal microbiota. *Enterococcus faecalis* is generally harmless for healthy individuals, but it can cause a diverse range of infections in immunodeficient or elderly patients with severe underlying diseases. In this study, we analysed the levels of intestinal translocation of indigenous enterococci in C57BL/6, CF-1 and CX3CR1^−/−^ mice upon clindamycin antibiotic-induced dysbiosis. We found that C57BL/6 was the most permissive model for enterococcal translocation and that initiation of *E*. *faecalis* translocation coincided with a threshold of enterococcal colonisation in the gut lumen, which once reached, triggered *E*. *faecalis* dissemination to deeper organs. We showed that the extent to which *E*. *faecalis* clinical strain VE14821 competed with indigenous enterococci differed between the C57BL/6 and CX3CR1^−/−^ models. Finally, using a simplified gnotobiotic model, we observed *E*. *faecalis* crossing an intact intestinal tract using intestinal epithelial cells as one route to reach the lamina propria. Our study opens new perspectives for assessing the effect of various immunodeficiencies and for investigating mechanisms underlying enterococcal translocation.

## Introduction

Enterococci are typical intestinal pathobionts. Although subdominant in the core intestinal microbiota and relatively harmless for healthy humans, under certain circumstances enterococci can cause infections such as bacteraemia, peritonitis, endocarditis, and urinary tract, wound, and device-related infections^1^. The successful survival of enterococci in the hospital environment can be attributed to multidrug resistance and pathogenic traits. *E*. *faecalis* and *E*. *faecium* are the *Enterococcus* species most commonly associated with infection and vancomycin resistance. Enterococci are now ranked as the third most common type of nosocomial pathogen, with *E*. *faecalis* accounting for 60 to 80% of enterococcal infections^2,3^. Despite increasing epidemiological evidence that intestinal domination by vancomycin-resistant enterococci (VRE) precedes human bloodstream infections^4–6^, it remains to be established whether *E*. *faecalis* blooming upon antibiotic treatment leads to translocation and subsequent human bloodstream infection.

Pioneering work using mouse models highlighted the effect of antibiotic treatments on enterococcal colonisation and translocation^7,8^. Enterococcal translocation has since been reported after antibiotic-induced dysbiosis, coinfection, severe inflammatory conditions, severe burn injuries and acute irradiation, and after disruption of intestinal mucosal barrier function in various mouse genetic backgrounds^8–16^. Miyazaki *et al*.^10^ showed that mice after treatment with antimicrobial agents and cyclophosphamide displayed increased susceptibility to VRE bacteraemia, leading to death in one-third of the mice following oral VRE inoculation. Amongst the 13 mouse lines tested under these conditions, C57BL/6 mice were identified as the strain most susceptible to VRE infection after oral inoculation. However, whether higher susceptibility of C57BL/6 mice correlates with a higher translocation efficiency remains to be established. Enterococcal translocation has also been investigated in some studies combining deficiency of mucosal immunity and intestinal dysbiosis^17,18^. CX3CR1^−/−^ mice have been shown to display increased levels of bacterial translocation to the mesenteric lymph nodes, with *Enterococcus* being the predominant genus^18^.

This study aimed to examine the interrelationships between *E*. *faecalis* overgrowth, spreading within the gut tissues and dissemination to extra-intestinal sites. Our previous studies, together with those of others, have shown that clindamycin treatment favours the overgrowth of enterococci in the lumen of the gastrointestinal tract of CF-1 mice^19–23^. Here, we examined the overgrowth and translocation of indigenous enterococci in C57BL/6, CF-1 and CX3CR1^−/−^ mice and that of an *E*. *faecalis* clinical isolate, VE14821, in clindamycin-treated C57BL/6 and CX3CR1^−/−^ mice. We also studied the passage of VE14821 from the lumen to the lamina propria in nonantibiotic-treated gnotobiotic mice.

## Results

### Clindamycin-treated C57BL/6 mice were more permissive for enterococcal translocation than CF-1 mice

**Figure 1:**
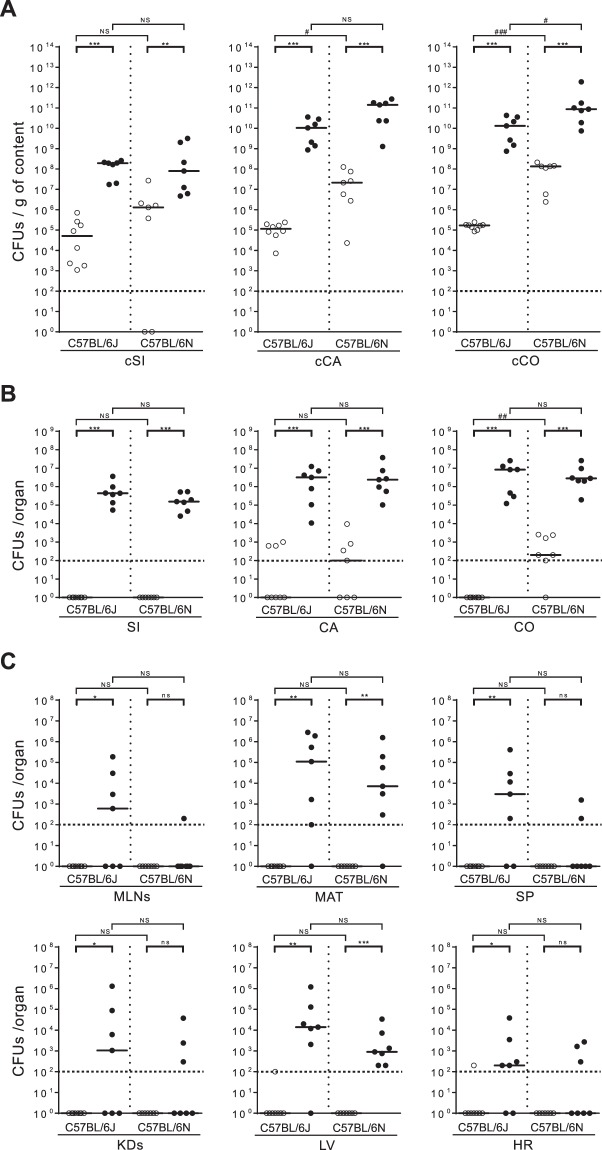
Effect of clindamycin on the levels of indigenous enterococci in the intestinal lumen and tissues, and translocation to organs in C57BL/6 mice. Each dot represents one mouse (n ≥ 7) and indicates the number of colony-forming units (CFUs) per gram of content (g of content) or per organ. The dotted line represents the detection limit. Horizontal bars represent the median values from two independent experiments. Enterococcal counts in the gut lumen (**A**) were conducted on luminal content from the small intestine (cSi), the caecum (cCA) and the colon (cCO) of C57BL/6J and C57BL/6N conventional mice that were untreated (◯) or treated for three consecutive days with clindamycin (●). Enterococcal counts in the gut tissues (**B**) were conducted on the small intestine (SI), the caecum (CA) and the colon (CO) from clindamycin-treated C57BL/6J and C57BL/6N conventional mice. Enterococcal counts in peripheral organs (**C**) were conducted on the mesenteric lymph nodes (MLNs), the mesenteric adipose tissue (MAT), the spleen (SP), the kidneys (KDs), the liver (LV) and the heart (HR) from clindamycin-treated C57BL/6J and C57BL/6N conventional mice. Statistical analysis was performed using the Mann–Whitney test. The asterisk (*) and hash (#) symbols indicate statistically significant differences between counts (*P < 0.05, **P < 0.01, ***P < 0.001; ^#^P < 0.05, ^##^P < 0.01, ^###^P < 0.001); ns, non-significant difference.

To evaluate the effect of clindamycin treatment on the levels of enterococcal translocation in C57BL/6 mice, we first assessed the induction of indigenous enterococcal overgrowth by clindamycin treatment in C57BL/6J and C57BL/6N mice, the two main substrains derived from the ancestral C57BL/6 mouse line. The levels of overgrowth in these substrains were then compared to the levels observed in CF-1 mice. The levels of viable enterococci in the contents of the lumens of the small intestine (cSi), caecum (cCA) and colon (cCO) were assessed by counting colony-forming units (CFUs). Untreated C57BL/6J and C57BL/6N mice were differentially colonised by indigenous enterococci. The median values of indigenous enterococci reached ∼5 × 10^4^ CFUs/g of cSI, ∼10^5^ CFUs/g of cCA and ∼10^5^ CFUs/g of cCO in C57BL/6J mice and ∼10^6^ CFUs/g of cSI, ∼10^7^ CFUs/g of cCA and ∼10^8^ CFUs/g of cCO in C57BL/6N mice (Fig. 1A). After three days of clindamycin treatment, the enterococcal population in the cSI, cCA and cCO of both C57BL/6 substrains increased, with the largest increase of ∼5-log being observed in the cCA and cCO of the C57BL/6J mice (Fig. 1A). As expected, enterococcal overgrowth occurred in the gut lumen of clindamycin-treated CF-1 mice (Supp. Fig. S1A). The level of enterococci in the cCA and cCO after clindamycin treatment was ∼10 to 100 times lower in the CF-1 mice than in the C57BL/6 mice substrains. These results confirmed that clindamycin treatment led to enterococcal overgrowth in the gut lumen of both C57BL/6 substrains, and also indicated that the baseline levels of enterococci were higher in the C57BL/6N mice than in the CF-1 mice, at least in the lumen of the caecum and colon. Enterococcal spreading within the gut in the small intestine (SI), the caecum (CA) and the colon (CO), and dissemination to extra-intestinal sites in the mesenteric lymph nodes (MLNs), the mesenteric adipose tissue (MAT), the spleen (SP), the kidneys (KDs), the liver (LV) and the heart (HR) were then assessed in both C57BL/6 substrains and in CF-1 mice. In the absence of clindamycin treatment, no or very few enterococci were detected in the gut tissues, lymphoid organs or deeper tissues, suggesting either that the levels of translocation of the indigenous enterococci in untreated mice were very low or that the host immune system was highly efficient (Fig. 1B,C, and Supp. Fig. S1B,C). In contrast, upon clindamycin treatment, enterococci in the C57BL/6 substrains reached median levels of 10^5^ CFUs in the SI, and 10^6^ CFUs in the CA and CO (Fig. 1B). Enterococci reached lower levels of ∼10^4^ CFUs in the SI and ∼5 × 10^5^ CFUs in the CO of CF-1 mice (Supp. Fig. S1B). Enterococcal translocation to extra-intestinal organs was detected in both C57BL/6 substrains, especially in the MAT and the LV, where levels reached 10^4^ to 10^5^ CFUs/organ and 10^3^ to 10^4^ CFUs/organ in the C57BL/6J and C57BL/6N mice, respectively (Fig. 1C). Translocation to the MLNs, SP, KDs and HR was barely detectable in the C57BL/6N mice, whereas median levels of ∼10^3^ CFUs/organ or just above were observed for the MLNs, KDs and SP, and median levels of just over 10^2^ CFUs/organ were observed for the HR in C57BL/6J mice. Although no statistically significant differences between the two substrains were observed under our conditions, these findings indicate that the levels of enterococcal translocation were higher in C57BL/6J than in C57BL/6N. Enterococci were detected in the MAT, LV and HR of clindamycin-treated CF-1 mice, with the LV being preferentially targeted (Supp. Fig. S1C). Together, these data show that clindamycin-induced enterococcal overgrowth in the gut lumen led to higher levels of enterococcal translocation in the C57BL/6 mice than in the CF-1 mice.

### Enterococcal translocation upon clindamycin treatment is no higher in CX3CR1^−/−^ mice than in CF-1 and C57BL/6 mice

**Figure 2:**
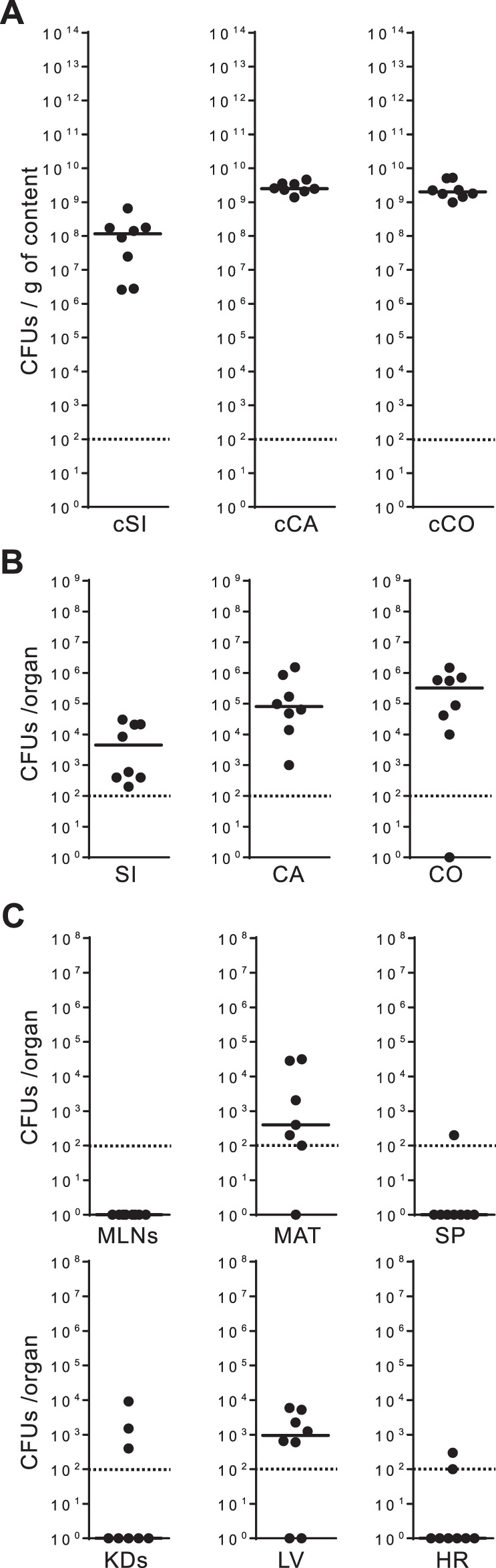
Translocation of indigenous enterococci in CX3CR1^−/−^ mice. Each dot (●) represents one CX3CR1^−/−^ mouse (n = 8) treated for three consecutive days with clindamycin and indicates the number of colony-forming units (CFUs) per gram of content (g of content) or per organ. The dotted line represents the detection limit. Horizontal bars represent the median values from two independent experiments. Enterococcal counts in the gut lumen (**A**) were conducted on luminal content from the small intestine (cSi), the caecum (cCA) and the colon (cCO). Enterococcal counts in the gut tissues (**B**) were conducted on the small intestine (SI), the caecum (CA) and the colon (CO). Enterococcal counts in the peripheral organs (**C**) were conducted on the mesenteric lymph nodes (MLNs), the mesenteric adipose tissue (MAT), the spleen (SP), the kidneys (KDs), the liver (LV) and the heart (HR).

Having established that enterococcal overgrowth in the gut lumen leads to a detectable level of translocation in clindamycin-treated C57BL/6 mice, we then analysed the level of enterococcal translocation in clindamycin-treated CX3CR1^−/−^ mice (C57BL/6N background). The level of enterococci in the gut lumen ranged between ∼10^8^ CFUs/g of cSI, and ∼10^9^ CFUs/g of cCA and cCO (Fig. 2A). The median levels reached in the gut tissues were ∼10^3^ CFUs in the SI, and ∼10^5^ CFUs in the CA and CO (Fig. 2B). Enterococcal translocation was detected in the MAT and in the LV, but the levels observed were either lower than or similar to those observed in the CF-1 and C57BL/6 mice (Fig. 2C). These results indicate that the levels of enterococcal translocation after clindamycin treatment were no higher in CX3CR1-deficient mice than in CF-1 and C57BL/6 mice.

### Enterococcal translocation requires a threshold level of enterococcal overgrowth in the lumen

**Figure 3:**
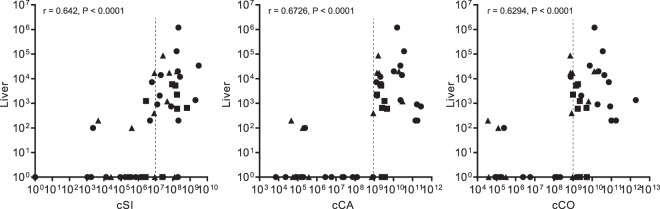
Correlations between enterococcal levels in the gut lumen and translocation to the liver. Spearman correlation analysis was used to evaluate the association between enterococcal levels in luminal content (X axis) from the small intestine (cSi), the caecum (cCA) and the colon (cCO), and in the liver (Y axis). Total enterococcal counts from CF-1 (▲), C57BL/6 (●) – including both the C57BL/6J and C57BL/6N substrains – and CX3CR1^−/−^ (■) mice are plotted. r indicates the Spearman’s rank correlation coefficient and P the p-value.

As our data suggested that the level of enterococcal intestinal overgrowth was a key determinant of enterococcal translocation, we performed a correlation analysis between the level of enterococci in the gut lumen and the levels of translocation to the LV in C57BL/6, CF-1 and CX3CR1^−/−^ mice. A positive correlation was revealed between higher levels of *E*. *faecalis* in the gut lumen and higher levels of translocation to the LV (Fig. 3). The distribution of the data indicates that intestinal translocation to the LV did not increase linearly with the level of enterococci in the intestinal lumen, but rather occurred once a threshold value had been reached. We estimated that these threshold values were ∼10^7^ CFUs/g in the cSI, and ∼10^9^ CFUs/g in the cCA and cCO.

### *E. faecalis* VE14821 colonises the lumen and spreads in gut tissues *in vivo*

With the ultimate goal of tracking the translocation of one exogenous strain *in vivo*, we performed invasion assays in HT-29 and T84 human colonic epithelial cells using eight *E*. *faecalis* strains of clinical origin (Table 1). Differences in the efficacy of invasion were observed between the eight strains at one hour post-infection (P < 0.01, ANOVA test) (Supp. Fig. S2A). The level of invasion of HT-29 cells with *E*. *faecalis* JH2-2 was below 0.005%, whereas that with OG1RF, V583, HH22, VE14518 and VE14818 was between 0.01% and 0.1%. Interestingly, invasion with VE14821 reached 0.5% (Supp. Fig. S2A, left panel). Similar levels of invasion with each of the strains were observed in the T84 cells, except that the levels of JH2-2 and VE14518 invasion were higher in the T84 cells than in the HT-29 cells. Invasion with VE14518 and VE14821 reached >0.3% in the T84 cells (Supp. Fig. S2A, right panel). Together, these data highlight the variability amongst *E*. *faecalis* strains regarding efficiency of internalisation within colonic epithelial cells. Of the eight candidate strains, VE14821 demonstrated the most enhanced invasive properties in both colonic epithelial cell types. Using T84 cells in a two-chamber transcytosis system, the transepithelial electrical resistance (TEER) through the cell monolayer was measured to evaluate monolayer integrity. The ability of VE14821 to cross the monolayer was compared with that of VE14518, OG1RF and JH2-2. Eight hours after infection, TEER was similar in the uninfected control monolayer and in the infected cells, regardless of the strain being used. These findings show that infection did not alter the integrity of the monolayer and suggest that the translocation involved a transcellular mechanism (Supp. Fig. S2B, left panel). The level of translocated bacteria recovered from the lower chamber indicated that all strains translocated across the cell monolayer with equal efficiency (Supp. Fig. S2B, right panel). These results show that, in addition to its propensity to invade colonic epithelial cells, *E*. *faecalis* VE14821 was also able to cross a cell monolayer. Based on all these results, VE14821 was selected for further *in vivo* investigation.

**Table 1:** *E*. *faecalis* strains used in this study.

Strains	Lab name	Origin	References
JH2-2	VE14000	Sepsis, UK, 1973	^37^
OG1RF	VE14001	Oral infection, USA, 1975	^38^
V583	VE14002	Blood, USA, 1987	^39^
HH22	VE18245	Urine, USA, 1981	^40^
VE14518		Deep pus, France, before 1999	This work
VE14818		Endocarditis, France, before 2005	This work
VE14819		Endocarditis, France, before 2005	This work
VE14821		Endocarditis, France, before 2005	This work
VE14821_cm_	VE18821	VE14821 transformed with the plasmid pJIM2246 conferring chloramphenicol resistance^41^	This work
VE14821_GFP_	VE18826	VE14821 transformed with the GFP-expressing plasmid pMV158-GFP conferring tetracycline resistance^42^	This work

**Figure 4:**
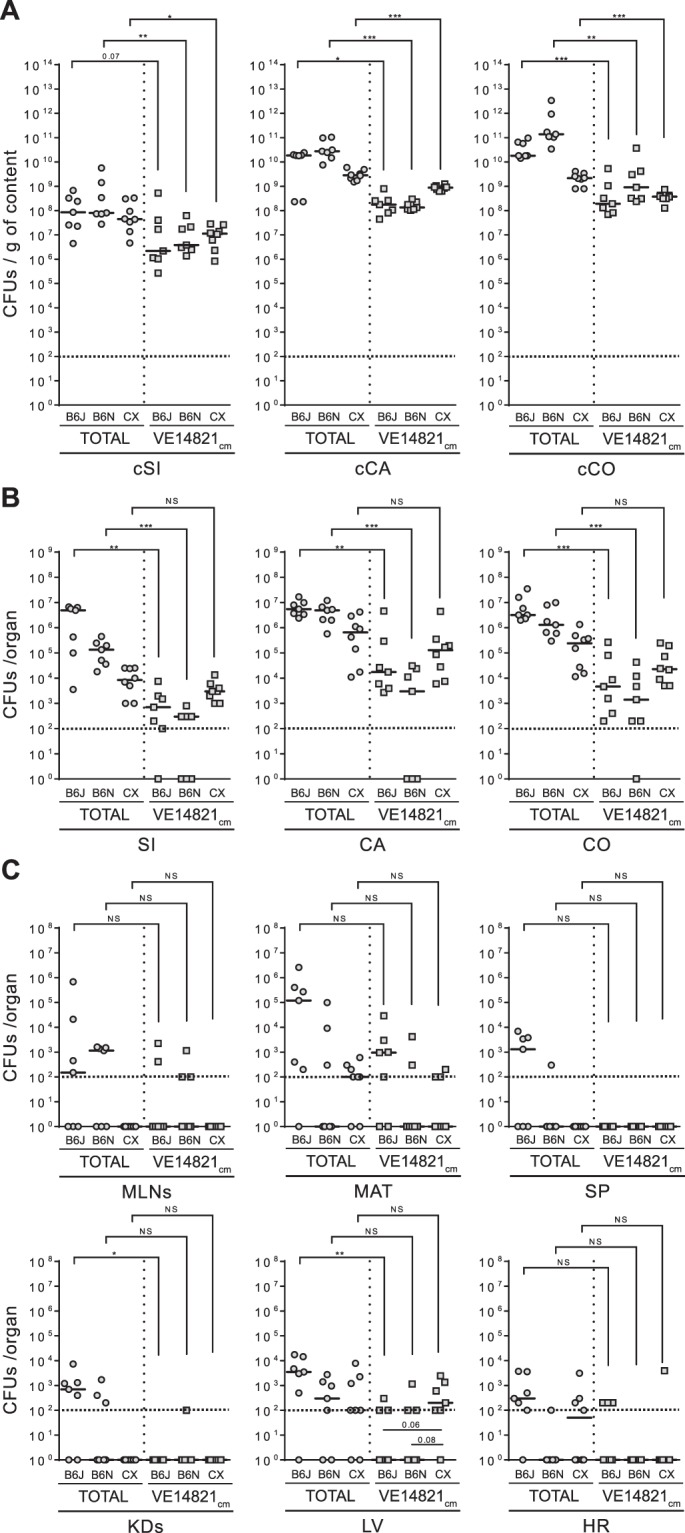
Translocation of *E*. *faecalis* VE14821_cm_ in clindamycin-treated C57BL/6 and CX3CR1^−/−^ mice. Total enterococcal counts () and VE14821_cm_ counts () were evaluated in clindamycin-treated C57BL/6J (B6J), C57BL/6N (B6N) and CX3CR1^−/−^ (CX) mice 13 hours after oral inoculation. Each dot represents one mouse (n ≥ 7) and indicates the number of colony-forming units (CFUs) per gram of content (g of content) or per organ. The dotted line represents the detection limit. Horizontal bars represent the median value from two independent experiments. Enterococcal counts in the gut lumen (A) were conducted on luminal content from the small intestine (cSi), the caecum (cCA) and the colon (cCO). Enterococcal counts in the gut tissues (B) were conducted on the small intestine (SI), the caecum (CA) and the colon (CO). Enterococcal counts in the peripheral organs (C) were conducted on the mesenteric lymph nodes (MLNs), the mesenteric adipose tissue (MAT), the spleen (SP), the kidneys (KDs), the liver (LV) and the heart (HR). Statistical analysis was performed using the Mann–Whitney test. Asterisks (*) indicate statistically significant differences between counts (*P < 0.05, **P < 0.01, ***P < 0.001); ns, non-significant difference.

C57BL/6J, C57BL/6N and CX3CR1^−/−^ mice were orally inoculated with VE14821 transformed with a plasmid conferring chloramphenicol resistance (VE14821_cm_). Enterococcal counts were analysed 13 hours after inoculation. As observed with the indigenous enterococci, VE14821_cm_ was mainly located in the distal regions of the gut lumen in each of the mice strains, with overgrowth levels of ∼10^8^ CFUs/g of cCA and cCO (Fig. 4A). VE14821_cm_ was found within the gut tissues at a median level of ∼10^3^ CFUs in the SI, ∼10^5^ CFUs in the CA and ∼10^4^ CFUs in the CO of CX3CR1^−/−^ mice; levels were 10-times higher than those observed in the corresponding gut tissues from the C57BL/6 mice (Fig. 4B). Although only low levels of VE14821_cm_ translocation to the deeper organs were observed all three types of mice, levels of translocation of VE14821_cm_ to the LV tended to be higher in the CX3CR1^−/−^ mice than in the C57BL/6 substrains (Fig. 4C). No significant differences in the levels of total indigenous enterococci were observed between C57BL/6J, C57BL/6N and CX3CR1^−/−^ mice, in either the absence or presence of VE14821_cm_, indicating that VE14821_cm_ affects neither the colonisation nor the translocation of indigenous enterococci (Mann-Whitney’s test; data not shown). VE14821_cm_ counts were lower than those for total enterococci in the lumen and the gut tissues of C57BL/6 mice, indicating that VE14821_cm_ did not compete with indigenous enterococci in the C57BL/6 substrains. Similar results were observed for the gut lumen in CX3CR1^−/−^ mice. In contrast, the levels of invasion by VE14821_cm_ and indigenous enterococci in the gut tissues were almost equal in the CX3CR1^−/−^ mice (Fig. 4B). In conclusion, the VE14821_cm_ strain was able to colonise the gut lumen and enter the intestinal tissue by 13 hours post-inoculation. Colonisation and spreading of VE14821_cm_ did not outcompete that of the indigenous enterococci but the extent to which *E*. *faecalis* clinical strain VE14821 competed with indigenous enterococci in the gut tissues differed between the C57BL/6 and CX3CR1^−/−^ models.

### Intestinal epithelial cells are one portal of entry for *E. faecalis* VE14821 to reach the lamina propria

**Figure 5:**
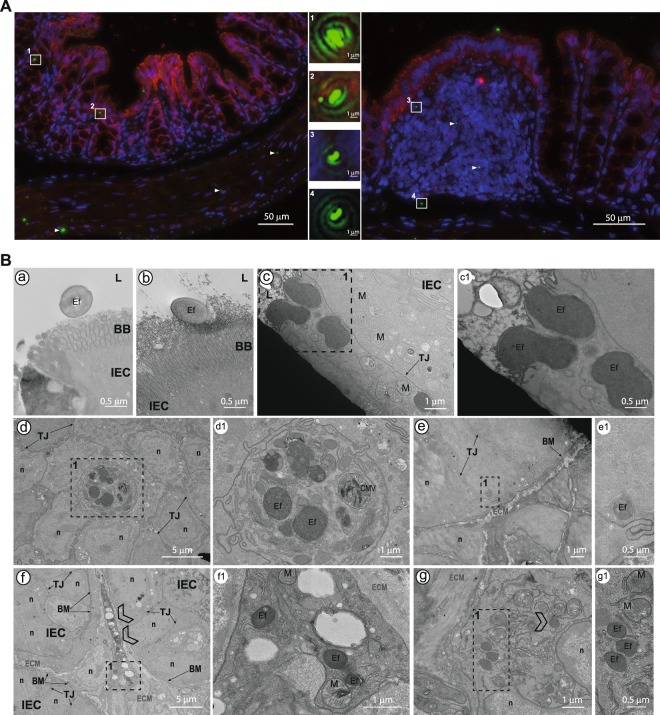
Intestinal crossing of *E*. *faecalis* VE14821_GFP_ in gnotobiotic C57BL/6J mice. (**A**) Immunofluorescence microscopy showing claudin-1 expression (red) and GFP-expressing *E*. *faecalis* (green) on a colonic section. Cell nuclei were stained with Hoechst. Bacteria, either from the epithelial villi (left panel) or from the colonic patch (right panel), are shown at higher magnification in boxed areas (1 to 4). The white triangles indicate additional VE14821_GFP_. (**B**) Transmission electron microscopy of the colon. Dotted windows indicate a region interest. Close-up images of these regions are shown in panels on the right of the original picture, with the correspondence between sets of images being indicated by a letter and a number. L: lumen; BB: brush border; Ef: *E*. *faecalis* VE14821_GFP_; IEC: intestinal epithelial cell; TJ: tight junction; n: nucleus; ECM: extracellular matrix; LP: lamina propria; M: mitochondria; BM: basal membrane. Scale bars are provided for each image.

To allow the intestinal crossing of VE14821 to be depicted, gnotobiotic mice were colonised by a VE14821_GFP_ strain expressing the green fluorescent protein (GFP). Thirteen hours after inoculation of germ-free C57BL/6J mice with VE14821_GFP_, VE14821_GFP_ was found at similar levels in the SI, in CA and in CO (Supp. Fig. S3A). In agreement with the well-established role of the microbiota in colonisation resistance^24^, this level was 10 to 1000-fold higher than that observed in conventional C57BL/6J mice. VE14821_GFP_ was localised by immunohistological examination of the colonic section, using classic double labelling of the claudin-1 tight junction protein and the nuclei to differentiate between mucosae from the submucosa and muscularis (Fig. 5A). The bacteria were well-distributed along the tissue and no particular site of passage could be detected. *E*. *faecalis* VE14821_GFP_ bacteria were present in colonic epithelial cells (Fig. 5A, areas 1 and 2), as well as in subepithelial patch domes (Fig. 5A, area 3), suggesting that *E*. *faecalis* was able to cross the gut barrier through either the epithelial cells or the follicle-associated epithelium covering the gut-associated lymphoid tissues. Interestingly, VE14821_GFP_ was also detected below the epithelium and lamina propria in the submucosa (Fig. 5A, left panel and area 4). The course of these events was then examined at higher magnification by transmission electron microscopy of the same colonic samples, and of sections from the SI and the CA. Based on observations of bacterial size and shape, the presence of the bacterial envelope, and the absence of characteristic mitochondrial crests, *E*. *faecalis* was found adhering to and entering into colonic epithelial cells (Fig. 5B, panels a, b and c), as well as cells in the CA and SI (Supp. Fig. S4A, panel a and Supp. Fig. S4B, panels a and b). During crossing of the villi, *E*. *faecalis* was found in other cell types (Fig. 5B, panel d). These other cells were not epithelial cells and did not have the dark appearance and large nucleus characteristic of intraepithelial lymphocytes (Supp. Fig. S3B). These *E*. *faecalis-*infected cells may have been phagocytic cells. Mucus drops in the goblet cells prevented detection of *E*. *faecalis* in this cell type (Supp. Fig. S3C). *E*. *faecalis* was observed close to the basal membrane before reaching the lamina propria (Fig. 5B, panel e and Supp. Fig. S4B, panel c). In each of these observations, the bacteria were cytosolic and not surrounded by a vacuole. *E*. *faecalis* was also detected in the lamina propria of the CO (Fig. 5B, panels f and g), the CA (Supp. Fig. S4A, panels b, c and d) and the SI (Supp. Fig. S4B, panel d). *E*. *faecalis* was found in the CA in granulocytes exhibiting light crystals in vesicles (Supp. Fig. S4A, panel d1). Finally, although part of the *E*. *faecalis* population seemed to be digested within the vacuoles (Supp. Fig. S4A, panels b2 and c), intact and free bacteria were found in the cell cytoplasm, as well as in the extracellular matrix, of the lamina propria (Fig. 5B, panel f1; Supp. Fig. S4A, panel b2 and Supp. Fig. S4B, panels d1 and d2). Our analysis demonstrates that intact intracellular bacteria were present in the epithelial cell layer and in the lamina propria. These intact bacteria were not surrounded by a vacuole. Intact extracellular bacteria were also detected in the extracellular matrix. Taken together these findings suggest that these bacteria are likely to be able to disseminate to deeper organs.

## Discussion

The mouse is a widely used model for studying intestinal microbiota, enteropathogens and the host immune response. However, the low efficiency of enterococcal translocation in mice in the absence of intensive treatments has not allowed studies to take full advantage of this model to decipher the mechanisms involved in *E*. *faecalis* pathogenesis originating from the gut. Here, we quantitatively assessed the relationship between clindamycin-induced overgrowth and translocation. Overgrowth led to spreading of enterococci in the CA and CO – with ∼10^5^ to 10^6^ CFUs per tissue – and then to subsequent dissemination to peripheral organs, especially to the MAT and LV to levels of ∼10^3^ to 10^5^ CFUs in C57BL/6 mice. C57BL/6 mice exhibited the highest level of enterococci in the gut lumen after clindamycin-treatment and were more permissive to enterococcal translocation than CF-1 and CX3CR1^−/−^ mice. These results definitively established the higher susceptibility of C57BL/6 mice to enterococci, strengthening previous data obtained by Miyazaki and colleagues^10^. Our findings show that *E*. *faecalis* translocation is only triggered once a threshold of overgrowth has been reached, rather than gradual translocation occurring conjointly with overgrowth in the lumen. In keeping with this conclusion, Chakraborty *et al*. recently reported that intestinal proliferation is required for translocation of enterococci upon ceftriaxone treatment^25^. Epidemiological studies have also reported an association between VRE intestinal domination and bloodstream infection^4–6^. The results of these previous studies, taken together with our finding support that enterococcal overgrowth may increase the risk for subsequent infection.

The CX3CR1 receptor and CX3CR1-expressing cells are involved in regulating bacterial translocation^18,26,27^. Steady state levels of bacterial translocation have been found to be high in CX3CR1^−/−^ mice, with *Enterococcus* being the predominant genus translocating to the MLNs, suggesting a role for CX3CR1-expressing cells in restricting translocation of bacteria from the microbiota^18^. Conversely, CX3CR1^hi^ cells have been shown to carry out trafficking of non invasive bacteria to the MLNs during antibiotic-induced dysbiosis, and this migration was found to be tightly regulated by the microbiota^27^. In antibiotic-treated mice, translocation of commensal bacteria – including that of *E*. *faecalis* – occurs through colonic goblet cell-associated antigen passages or GAPs; however, this pathway has been shown to be abolished in the absence of CX3CR1^26^. Taking into account the important role played by CX3CR1^+^ cells in transporting commensal bacteria to the MLNs, our findings suggest that abolition of CX3CR1 expression restricts the level of enterococci in the gut lumen, thereby limiting *E*. *faecalis* translocation to extra-intestinal sites after antibiotic therapy.

We found that the clinical isolate VE14821 was able to colonise the gut lumen and enter into the intestinal tissue of both C57BL6 and CX3CR1^−/−^ mice. In the CX3CR1^−/−^ mice, VE14821 competed with indigenous enterococci in the gut tissues. However, translocation of VE14821 was inefficient, as the optimal threshold of enterococci in the intestinal lumen had not been reached. VE14821 is a strain of human origin and may not be as well-adapted to the murine gut as the indigenous enterococci, which could have delayed or limited VE14821 overgrowth. Our in-depth analysis of the gut tissues of VE14821-inoculated gnotobiotic C57BL/6 mice provided clear evidence of the passage of VE14821 from the lumen and its internalisation in the epithelial cells before reaching the lamina propria. Once at the lamina propria, *E*. *faecalis* was found in a free state outside cells in the extracellular matrix, a finding consistent with reports indicating that enterococci have the ability to bind extracellular matrix proteins^28–30^. Remarkably, *E*. *faecalis* was also found intracellularly in a free state in the cytosol, supporting findings from previous studies indicating that bacteria are able to hijack lysosomal degradation pathways^31,32^. These observations are reminiscent of those described in the early study of Wells *et al*.^7^ in which bacteria resembling *E*. *faecalis* always appeared to be within the epithelial cells, and support the idea that intestinal epithelial cells provide a portal of entry for enterococci via a transcellular mechanism.

In conclusion, we established that intestinal translocation of enterococci requires a threshold level of enterococcal overgrowth in the lumen and depicted the passage of *E*. *faecalis* through the intestinal mucosa, opening new perspectives for investigating mechanisms underlying enterococcal translocation. Together with epidemiological data and our study, determination of the overgrowth threshold in severely ill and immunodeficient patients may be an important strategy for monitoring their risk of developing enterococcal infection.

## Methods

### Bacterial strains

The *E*. *faecalis* strains used in this study are listed in Table 1. *E*. *faecalis* was routinely grown in brain heart infusion (BHI) medium at 37 °C without aeration. Strains VE14821_cm_ and VE14821_GFP_ were selected and grown in BHI medium supplemented with 10 μg/ml of chloramphenicol or 4 μg/ml of tetracycline, respectively.

### Cell lines

The human T84 colonic epithelial cell line (ECACC 88021101) was grown in a 1:1 mixture of Dulbecco’s modified Eagle’s medium (DMEM) and Ham’s F12 medium with 10% foetal bovine serum (FBS) and 2 mM glutamine. The human HT-29 colonic epithelial cell line (ECACC 91072201) was grown in Roswell Park Memorial Institute medium (RPMI 1640) with 10% FBS, 2 mM glutamine, 0.1 mM non-essential amino acids and 1 mM sodium pyruvate. All cells were incubated at 37 °C in a 5% CO_2_ atmosphere.

### Ethics statement

Experiments on conventional animals were approved by the local ethics committee, the COMETHEA (“Comité d’Ethique en Expérimentation Animale du Centre INRA de Jouy en Josas et AgroParisTech”), under the registration numbers 15_08 and 15_19, and by the French Ministry of Higher Education and Research (# 00680.01 and APAFIS # 480 -2015041518048149v1). Experiments on germ-free animals were approved by the local ethics committee, under the registration number 17_02, and by the French Ministry of Higher Education and Research (APAFIS # 565-2015042116189612v3). All animal experiments were performed in accordance with European directive 2010/63/EU.

### Animal studies

All experiments were conducted on 9 to 12-week-old adult male mice. All C57BL6/J and C57BL6/N conventional mice were purchased from Charles River (France). CF-1 mice, originally purchased from Envigo (Indianapolis, USA), were raised at the CDTA-CNRS (Orléans, France). Mice were housed in groups of four animals per filter-top cage and fed with irradiated food and autoclaved water *ad libitum*, in line with animal welfare guidelines and to minimise coprophagia The animal house was maintained on a 12-hour light/dark cycle. All animals were adapted to the environment of the local animal facilities (IERP, INRA, Jouy-en-Josas) for at least one week prior to study. CX3CR1^−/−^ conventional mice were generated by C. Combadiere *et al*.^33^ and purchased from Taconic Biosciences (USA): the CX3CR1^−/−^ mouse line had been backcrossed to C57BL/6NAi for 10 generations and then backcrossed to C57BL/6NTac for five generations (Model #4167). CX3CR1^−/−^ mice were bred after embryo transfer in FVB*/*N mice to eliminate *Chilomastix* endoparasites and then housed under specific pathogen-free conditions in our local animal facility (IERP, INRA, Jouy-en-Josas). All germ-free mice were obtained locally from the germ-free rodent breeding facility Anaxem (INRA, Micalis Institute, France). Experiments on germ-free C57BL/6J mice were conducted on adult (11-week-old) males. *E*. *faecalis* strains were collected by centrifugation 1 h after they had reached the stationary phase. Bacterial cells were washed twice with phosphate-buffered saline (PBS) 1X and stored at −80 °C. For translocation assays, 9 to 12-week-old conventional CF-1, C57BL/6J, C57BL/6N and CX3CR1^−/−^ male mice received a subcutaneous dose of 1.4 mg/day of clindamycin for three days. When required, one day later, ∼1 × 10^10^ CFUs of VE14821_cm_ were orally administered by gavage using a feeding tube. The gnotobiotic mice received no antibiotic treatment. Eleven-week-old C57BL/6J axenic males received ∼1 × 10^10^ CFUs of *E*. *faecalis* VE14821_GFP_, orally administered by gavage using a feeding tube. Luminal contents and organs were collected either after antibiotic treatment (when required) or 13 hours after *E*. *faecalis* inoculation. MLNs, MAT, SP, KDs, LV and HR were homogenised in PBS immediately after collection. The SI, CA and CO were opened and washed five times in DMEM. To kill all remaining extracellular bacteria in the gut lumen, gut tissues were incubated for 2 hours in DMEM containing 50 μg/ml teicoplanin, 20 μg/ml daptomycin and 250 μg/ml amoxicillin. After five washes in DMEM to remove the antibiotics, gut tissues were homogenised in PBS. Serial dilutions of organ homogenates were transferred to plates containing bile esculin azide agar (BEA Agar, BK158HA from Biokar diagnostics), a selective medium for monitoring indigenous enterococci, or to BEA supplemented with chloramphenicol or tetracycline to determine VE14821_cm_ or VE14821_GFP_ counts, respectively. Counts were determined 42–48 hours after tissue plating and partial sequencing of the 16S rRNA genes in samples of isolates confirmed successful selection of enterococci in >94% of cases (unpublished data). Serial dilutions of the inoculum were also transferred to plates as a control for determining *E*. *faecalis* numbers in the inoculate. Statistical analyses to compare the levels of bacteria translocated to the various tissues were performed using the Mann–Whitney test and the GraphPad Prism software (Version 6.03).

### Analysis of correlation

The relationship between enterococcal counts in the gut lumen and those in the LV was analysed using Spearman’s correlation and the GraphPad Prism software.

### Cell invasion assay

Cell invasion experiments were performed as previously described^34^. Briefly, *E*. *faecalis* strains were grown to an OD_600_ of 0.6 and used at a multiplicity of infection (MOI) of 50. After one hour of contact, extracellular bacteria were killed using an antibiotic cocktail containing 150 µg/ml gentamycin, 25 µg/ml linezolid and 250 µg/ml amoxicillin for the JH2-2, OG1RF, VE14518 and VE14818 strains, or an antibiotic cocktail containing 50 μg/ml teicoplanin, 20 μg/ml daptomycin and 250 μg/ml amoxicillin for the HH22, V583, VE14819 and VE14821 strains. The antibiotic concentrations chosen were based on those giving no extracellular bacteria remaining upon plating of infected cell supernatant for at least one member of each group shown above. Cells were lysed one hour post-infection with 0.5% Triton X-100, and intracellular bacteria were counted after transfer to BHI agar plates. Percentage invasion was determined as the ratio of intracellular bacteria to the number of bacteria in the initial inoculum. Experiments were performed in triplicate wells for at least four independent assays. Results were analysed by a nonparametric one-way analysis of variance (ANOVA). Further statistical analysis or post-hoc comparisons were done using the Tukey test and the GraphPad Prism software.

### T84 translocation assay

Translocation experiments were performed as previously described^35,36^. Briefly, T84 cells were seeded in a 12-well Transwell system with 3 μm-pore size polycarbonate membranes (Corning). Formation and integrity of the cell monolayer was monitored by measuring the TEER with the Millicell® Electrical Resistance System (ERS-2, Millipore). Translocation assays were performed when the TEER was stable and had reached 1500 Ω or higher, which usually occurred within 14 days. The day before the experiment, the cells were washed and fresh medium was added. Cells were then infected by adding the bacteria to the upper chamber (8 × 10^7^ CFUs in 50 μl). Viable bacteria in the lower chamber were counted at four and eight hours post-infection after serial dilutions of an aliquot were transferred to BHI agar plates. Experiments were performed in duplicate wells for at least four independent assays. The difference in TEER between the infected cells and an uninfected control was analysed using the unpaired Student’s t-test. Differences in the levels of translocation between the strains were tested by a non-parametric one-way ANOVA using the GraphPad Prism software.

### Histology and immunostaining

Flushed intestines were fixed in 4% paraformaldehyde, dehydrated and then embedded in paraffin. Slide-mounted sections were prepared with a rotary microtome. Immunostaining was performed according to standard protocols. The sections were deparaffinised in xylene and rehydrated using a descending alcohol series. Antigen retrieval was performed by incubating the sections in 10 mM sodium citrate, pH 6.5, in a heated water bath at 97 °C for 40 min. Following a further one-hour incubation at room temperature in 0.4% Triton X-100 in PBS, the sections were transferred to a blocking solution (BSA 1% in PBS buffer). Anti-GFP antibodies were used to reduce green autofluorescence emitted from the tissue and allow detection of GFP-expressing VE14821. Sections were incubated overnight at 4 °C with primary antibodies: mouse anti-claudin 1 (Invitrogen, diluted 1:100) and rabbit anti-GFP (Roche, diluted 1:200). Fluorescence-labelled anti-mouse DyLight 488 (Thermo Fisher Scientific, diluted 1:500) and anti-rabbit A568 (Thermo Fisher Scientific, diluted 1:2000) were used as secondary antibodies. Nuclei were stained with Hoechst 33342 (Sigma). Sections were scanned with a digital slide scanner (Pannoramic Scan 3DHistech).

### Electron microscopy

All samples were treated using a protocol similar to that described previously^34^. Briefly, individual intestinal segments from the SI, CA and CO were ligatured on both sides. Fixation was performed by injecting within the closed segment a 1:1 mix of 4% glutaraldehyde in 0.2 M Na cacodylate buffer, pH 7.5, followed by incubation for 3 h at room temperature. Thin sections (70 nm) were collected onto 200 mesh cooper grids, and counterstained with lead citrate. Grids were examined with Hitachi HT7700 electron microscope operated at 80 kV (Elexience – France), and images were acquired with a charge-coupled device camera (AMT). Microscopic observations were performed on samples from two independent experiments, each performed on one mouse.

## Supplementary information

Supplemenatry info
