# Supplementary material for: Intestinal translocation of enterococci requires a threshold level of enterococcal overgrowth in the lumen
Source: Sci Rep. 2019 Jun 20;9:8926. doi: 10.1038/s41598-019-45441-3 (PMC6586816; doi:10.1038/s41598-019-45441-3)

Intestinal translocation of enterococci requires a threshold level of enterococcal overgrowth in the lumen

Cristel Archambaud[a,*], Aurélie Derré-Bobillot[a], Nicolas Lapaque[a], Lionel Rigottier-Gois[a] and Pascale Serror[a,*]

[a]Micalis Institute, INRA, AgroParisTech, Université Paris-Saclay, 78350 Jouy en Josas, France

[*]Corresponding authors: cristel.archambaud@inra.fr; pascale.serror@inra.fr

Tel : +33134652083

Fax : +33134652065

Running title: Gastro-intestinal tract translocation of *E. faecalis*

Keywords: *Enterococcus*, intestinal translocation

Supporting information

Supp. Figure S1. Analysis of enterococcal translocation in CF-1 mice. Each dot represents one CF-1 conventional mouse that was untreated (○) or treated for three consecutive days with clindamycin (●) and indicates the number of colony-forming units (CFUs) per gram of content (g of content) or per organ (n = 8, except for the luminal content of untreated mice in S1A, where n = 5). The dotted line represents the detection limit. Horizontal bars represent the median values from two independent experiments. Enterococcal counts in the gut lumen (A) were conducted on luminal content from the small intestine (cSi), the caecum (cCA) and the colon (cCO). Enterococcal counts in the gut tissues (B) were conducted on the small intestine (SI), the caecum (CA) and the colon (CO). Enterococcal counts in the peripheral organs (C) were conducted on the mesenteric lymph nodes (MLNs), the mesenteric adipose tissue (MAT), the spleen (SP), the kidneys (KDs), the liver (LV) and the heart (HR). Statistical analysis was performed using the Mann–Whitney test. Asterisks (*) indicate statistically significant differences between counts (*P < 0.05, **P < 0.01, ***P < 0.001); ns, non-significant difference.

Supp. Figure S2. Enterococcal invasion and translocation assays in intestinal cells. (A) Invasion assays. Human colonic epithelial cells (HT-29 and T84) were infected with eight *E. faecalis* strains at a multiplicity of infection of (MOI) of 50. Percentage invasion was determined after one hour as the ratio of intracellular bacteria to the number of bacteria in the initial inoculate. (B) Translocation assay. T84 cells were infected with four *E. faecalis* strains (JH2-2, OG1RF, VE14518 and VE14821). The integrity of the cell monolayer was evaluated by measuring transepithelial electrical resistance (TEER) for eight hours post-infection. The TEER of infected

cells was measured (left panel) and compared to that of uninfected cells (control). Translocation of each strain was evaluated by determining the number of enterococcal colony-forming units (CFUs) in the lower chamber of the transwell system (right panel). Data are expressed as the mean and standard error of the mean (SEM) of at least four independent experiments. Statistical analysis was performed using a non-parametric one-way analysis of variance (ANOVA). Asterisk (*) and hash (#) symbols indicate statistically significant differences between values (**$P < 0.01$, ***$P < 0.001$; # $P < 0.05$, ## $P < 0.01$). NS, non-significant difference.

Supp. Figure S3. Translocation of *E. faecalis* VE14821$_{GFP}$ in gnotobiotic C57BL/6J mice. (A) Total VE14821$_{GFP}$ counts (□) in the gut tissues and in the peripheral organs of C57BL/6J gnotobiotic mice 13 hours after oral inoculation with VE14821$_{GFP}$. Each dot represents one mouse (n = 4) and indicates the number of colony-forming units (CFUs) per organ. The dotted line represents the detection limit. Horizontal bars represent the median value. Counts in the gut tissues were conducted on the small intestine (SI), the caecum (CA) and the colon (CO). Counts in the peripheral organs were conducted on the mesenteric lymph nodes (MLNs), the mesenteric adipose tissue (MAT), the spleen (SP), the kidneys (KDs), the liver (LV) and the heart (HR). (B) Transmission electron microscopy showing an intraepithelial lymphocyte. (C) Transmission electron microscopy showing a goblet cell (GC) between two intestinal epithelial cells (IEC). L: lumen; BB: brush border. Scale bars are provided for both images.

Supp. Figure S4. Crossing of *E. faecalis* VE14821$_{GFP}$ into the caecum and small intestine of gnotobiotic C57BL/6J mice. Transmission electron microscopy of the caecum (A) and small

intestine (B). Dotted windows indicate regions of interest. Close-up images of these regions are presented in the panels to the right of the original picture, with the correspondence between sets of images being indicated by a letter and a number. The double arrow indicates the host cell membrane. L: lumen; BB: brush border; Ef: *E. faecalis* VE14821$_{GFP}$; IEC: intestinal epithelial cell; TJ: tight junction; n: nucleus; ECM: extracellular matrix; LP: lamina propria; M: mitochondria; BM: basal membrane. Scale bars are provided for each image.

Supp. Figure S1

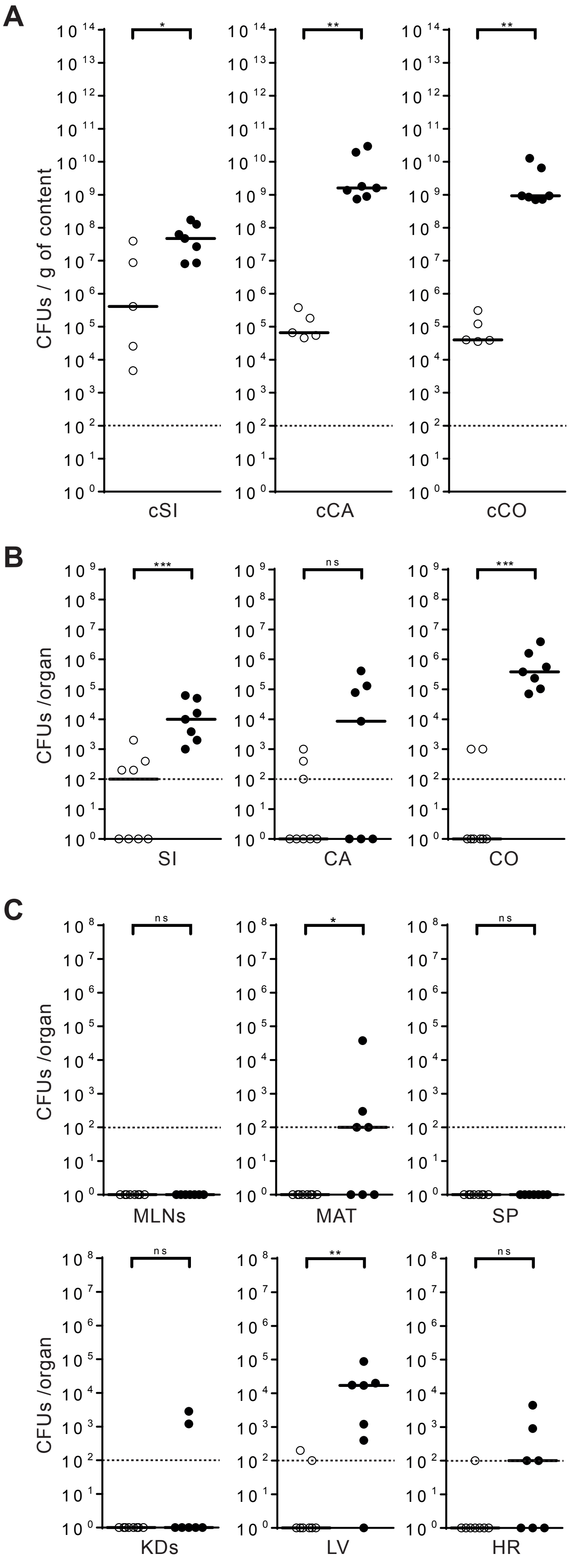

Supp. Figure S2

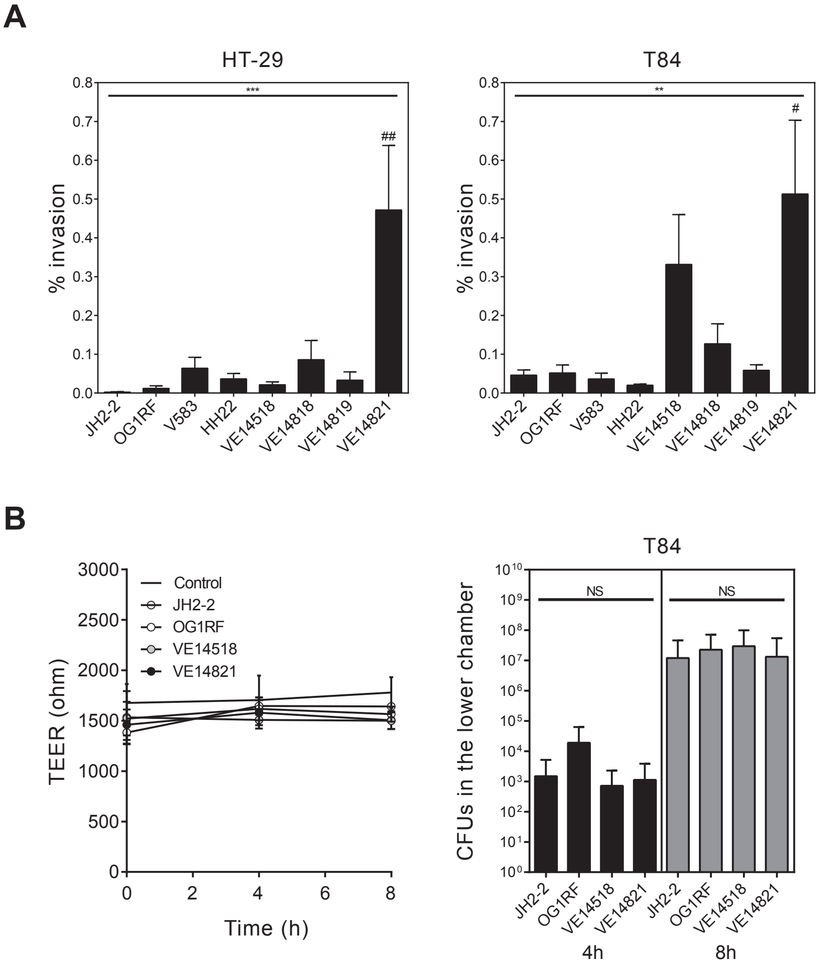

Supp. Figure S3

A

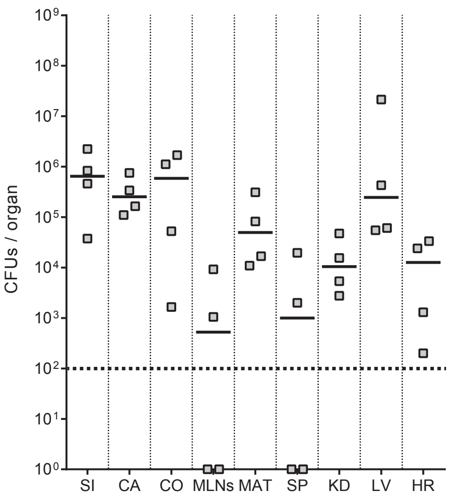

B

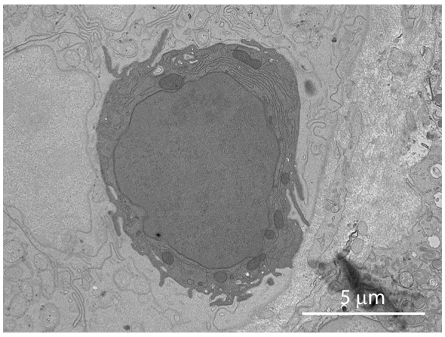

C

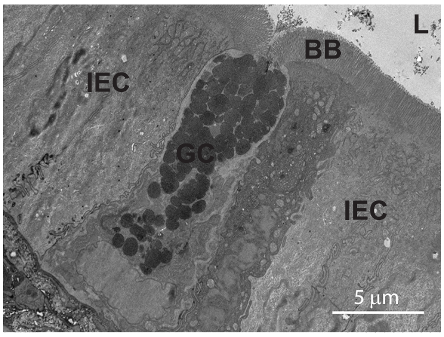

Supp. Figure S4

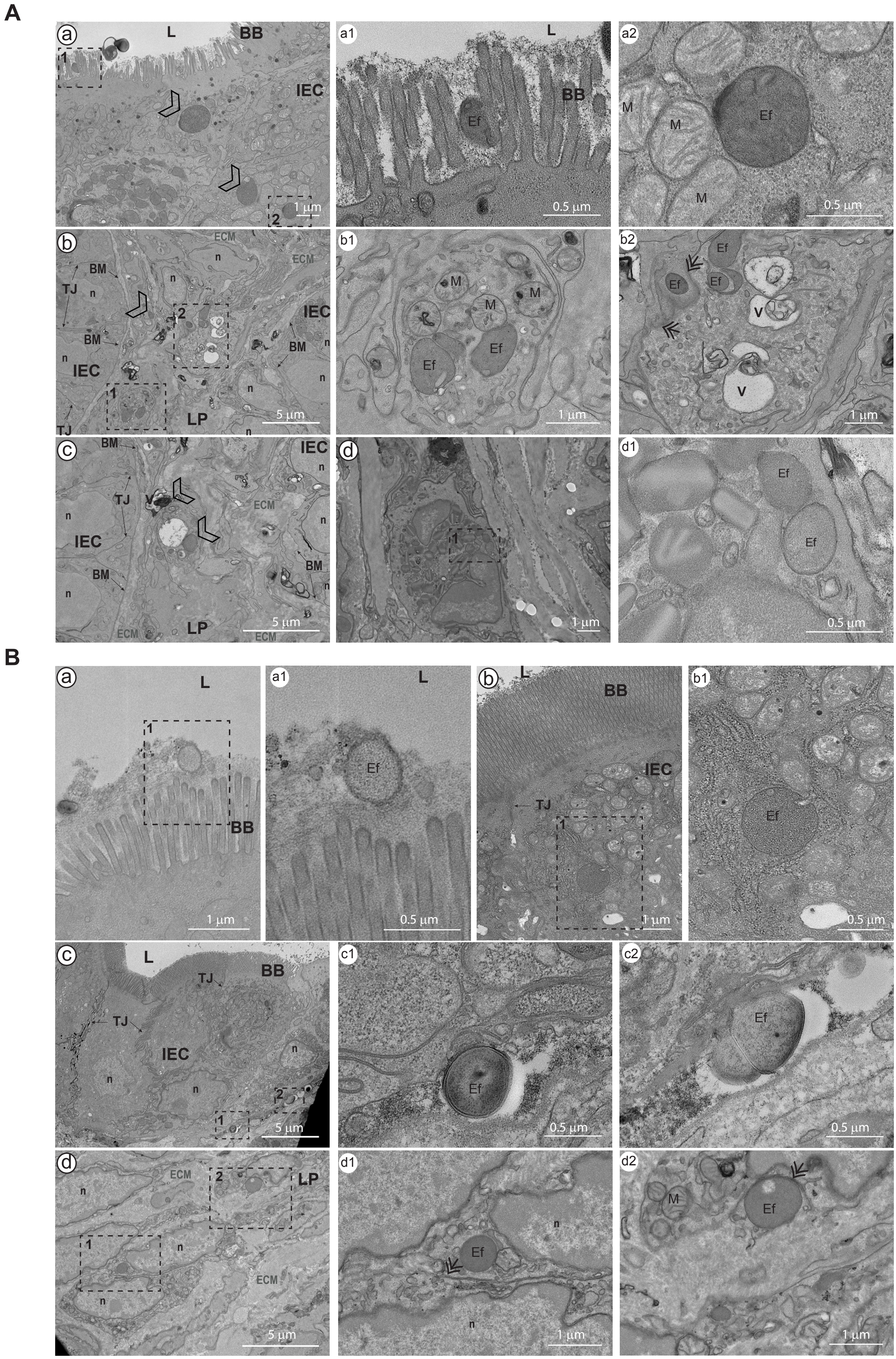

Supplement: Supplementary file 1 — Supplemenatry info [file 41598_2019_45441_MOESM1_ESM.pdf]
